# Impact of anti-arrhythmic drugs and catheter ablation on the survival of patients with atrial fibrillation: a population study based on 199 433 new-onset atrial fibrillation patients in the UK

**DOI:** 10.1093/europace/euac155

**Published:** 2022-09-15

**Authors:** Sheng-Chia Chung, Alvina Lai, Gregory Y H Lip, Pier D Lambiase, Rui Providencia

**Affiliations:** UCL Institute of Health Informatics Research, University College London, 222 Euston Rd, London NW1 2DA, UK; UCL Institute of Health Informatics Research, University College London, 222 Euston Rd, London NW1 2DA, UK; Liverpool Centre for Cardiovascular Science, University of Liverpool and Liverpool Heart & Chest Hospital, Liverpool, UK; Department of Clinical Medicine, Aalborg University, Aalborg, Denmark; UCL Institute of Cardiovascular Science University College London, London, UK; St Bartholomew’s Hospital, Barts Health NHS Trust, West Smithfield, London, UK; UCL Institute of Health Informatics Research, University College London, 222 Euston Rd, London NW1 2DA, UK; St Bartholomew’s Hospital, Barts Health NHS Trust, West Smithfield, London, UK

**Keywords:** Rhythm control, Arrhythmia, Mortality, Pulmonary vein isolation, Anti-arrhythmic agents

## Abstract

**Aims:**

Utilizing real-world UK data, we aimed to understand: (i) whether anti-arrhythmic drugs and catheter ablation are effective in improving the survival of atrial fibrillation (AF) patients and (ii) which rhythm control option produces better results for the whole AF population and for specific groups of patients, stratified by age, sex, and history of heart failure.

**Methods and results:**

We identified 199 433 individuals (mean age at diagnosis 75.7 ± 12.7 years; 50.2% women) with new-onset AF diagnosis in nationwide electronic health records linking primary care consultation with hospital data and death registry data from 1998 to 2016. We investigated the survival and causes of death of new-onset AF patients receiving vs. not-receiving rhythm control therapies. During a median follow-up of 2.7 (0.7–6.0) years, we observed a significantly lower mortality in patients receiving rhythm control [multivariate-adjusted hazard ratio (HR) = 0.86, 95% confidence interval (CI) 0.84–0.88]. Pulmonary vein isolation was associated with a two-third significant mortality reduction compared with no rhythm control (HR = 0.36, 95% CI 0.28–0.48), flecainide with 50% reduction (HR = 0.52, 95% CI 0.48–0.57), and propafenone and sotalol with reduction by a third (HR = 0.63, 95% CI 0.50–0.81, 0.71, 95% CI 0.68–0.74, respectively). Amiodarone showed no survival benefit in individuals <70 years (HR = 0.99, 95% CI 0.97–1.02). Otherwise, the effect of rhythm control on survival did not differ by age, sex, nor history of heart failure.

**Conclusion:**

Among individuals with new-onset AF, favourable survival was observed for patients receiving rhythm control treatment. Among different rhythm control strategies, pulmonary vein isolation showed the most pronounced survival benefit.

## Graphical Abstract

**Graphical Abstract:**
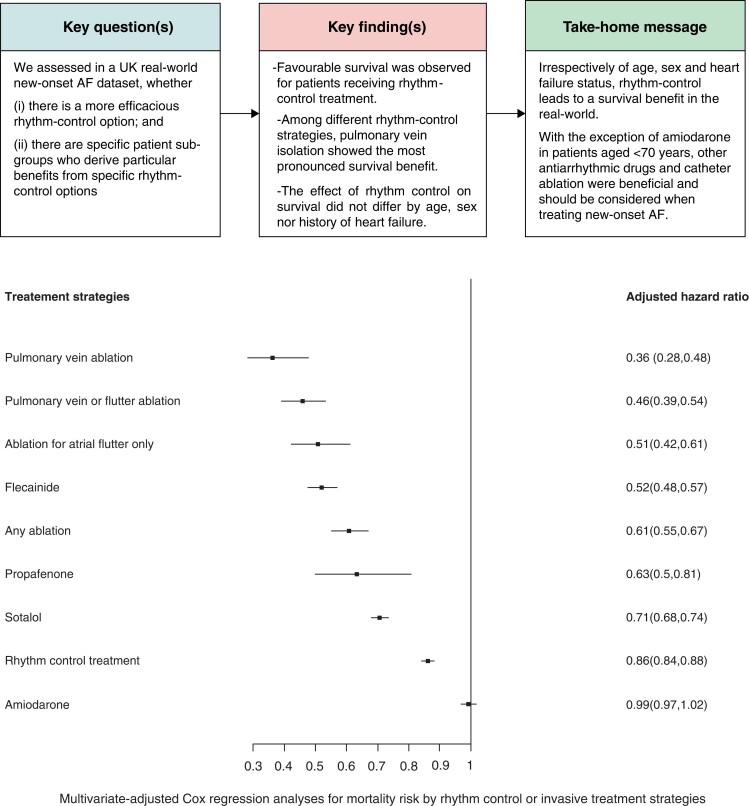

**What’s new?**

- We present real-world UK data on different rhythm control strategies for the management of atrial fibrillation.
- Pulmonary vein isolation was the strategy showing the most pronounced survival benefit.
- Among drugs, flecainide was associated with greater mortality reduction.
- Amiodarone showed no survival benefit in individuals aged <70.
- For all other rhythm control options, the effect on survival did not differ by age, sex, nor history of heart failure.

## Introduction

Atrial fibrillation (AF) is the most frequent cause of sustained arrhythmia in clinical practice affecting at least a third of the population in their lives.^1^ A total of 1.5 M people in the UK have been diagnosed with AF, and hundreds of thousands more remain undiagnosed.^2^ Appropriate characterization and holistic management of patients with AF is required as per guideline recommendations for the management of AF.^3^ Guidelines recommend the use of the Atrial Fibrillation Better Care pathway, which consists of three main pillars: A, avoid stroke (with anticoagulants); B, better symptom control management, with patient-centred decisions on rhythm vs. rate control decisions; and C, cardiovascular and comorbidity risk optimization.^4^

Whilst the advantages of anticoagulation in reducing thromboembolic risk and mortality^5^ have been widely accepted, the use and benefit of strategies to rate or rhythm control AF patients have been a matter of debate. Pooled evidence from trials suggests a survival benefit for AF ablation,^6^ and a recent clinical trial (EAST-AFNET4)^7^ suggested that patients with a recent diagnosis of AF who were treated with early rhythm control using anti-arrhythmic drugs or catheter ablation have better survival. Several drugs were used to control the irregular heart rhythm (such as propafenone, flecainide, and amiodarone). Nonetheless, data on the comparison of rhythm control strategies are sparse. Hence, the following questions remain unanswered: is there a more efficacious rhythm control option? Are there specific subgroups of patients who derive particular benefits from specific treatment options?

Nationwide electronic health records (EHRs) provide a unique opportunity to investigate the prognosis of AF management strategies in the general population receiving usual care. In the current study, utilizing real-world UK data, we specifically aim to: (i) understand whether heart rhythm drug treatments or catheter ablation are more effective in improving survival and (ii) which treatment option produces better results for the whole AF population and for specific groups of patients, stratified by age, sex, and history of heart failure.

## Methods

### Data sources

The study applied population linked EHRs, linking the Clinical Practice Research Datalink data of primary care consultation with hospital data (Hospital Episodes Statistics) and death registry data (Office for National Statistics, ONS).^8^ The data are generally representative of the age, gender, and geographic distribution of the UK population.^9^ Previous validation studies of the UK EHR showed high quality and completeness of clinical information recorded in the data.^8,9^ The Medicines and Healthcare products Regulatory Agency Independent Scientific Advisory Committee [17_205], under Section 251 of the National Health Service (NHS) Social Care Act 2006 approved the data use for the present study. The study followed the Reporting of studies Conducted using Observational Routinely collected health Data recommendations.^10^

### Study population and design

The study population was 199 433 patients admitted to a hospital with a primary diagnosis of AF from 1 January 1998 to 31 May 2016. We set the study entry date to the date of the initial AF. Patients were followed up until study endpoint, death, transfer out of the general practice, last day of the general practice data collection, or end of the study period (31 May 2016).

### Treatment and comparison

The treatment group of rhythm control treatment included treatment with anti-arrhythmic drugs, including amiodarone, sotalol, flecainide, propafenone, and catheter ablation. The definition of anti-arrhythmic drugs and ablation in health records are summarized in the Supplementary*Appendix* (see Supplementary material online, *Tables S1* and *S2*). The comparison group was individuals with AF not receiving rhythm control treatment.

### Primary outcome

The primary endpoint was all-cause mortality. We identified death, date of death, and causes of death from the ONS records. The cumulative case-fatality proportion was defined as the per cent of deaths among all incidence AF cases between treatment groups.

### Baseline covariates

We used the index of multiple deprivation 2015 quintile to describe socioeconomic status, with a higher quintile representing the more deprived areas.^11^ For new-onset AF cases, we studied 18 common chronic conditions associated with AF reported in the literature^12^ or with high prevalence observed in the study cohort, such as hypertension, diabetes, valvular disease, hyperthyroidism, angina, ischaemic heart disease (including unstable angina and acute myocardial infarction), heart failure, stroke, transient ischaemic attack, pulmonary embolism, deep vein thrombosis, peripheral artery disease, supraventricular tachycardia, ventricular tachycardia, cancer, chronic kidney disease, chronic obstructive pulmonary disease, and dementia. Patients without a diagnosis were assumed to be free from that condition. We reported the proportion of individuals with a diagnosis recorded in their primary care or hospital admissions, before their initial diagnosis of AF. CHA_2_DS_2_-VASc score was calculated (congestive heart failure, hypertension, age, diabetes, stroke or transit ischaemic attack, vascular disease, and sex).^13^ Diagnosis code lists for each condition were adapted from the CALIBER code repository^14^ (see Supplementary material online, *Table S3*).

### Statistical analyses

Baseline characteristics were presented in treatment groups. We reported frequencies (%) for categorical data and means with standard deviation for continuous data, and *χ*^2^ and *t*-tests were used to examine the difference between sex, age categories (≤50, 50–70, >70), and socioeconomic categories.

The difference in the rate of primary endpoints was compared by adjusted Kaplan–Meier curves between rhythm control groups and by a priori population subgroups (categories of age, sex, history of heart failure). For subgroup analyses by multiple deprivation status at baseline, we used the Kaplan–Meier estimation to evaluate the proportional hazard assumption. When appropriate, the Cox regression model was applied to estimate the hazard ratios (HRs). To control for varying therapy starting times of the rhythm control therapies, we modelled rhythm control as a time-varying variable. The Cox models were adjusted by age, sex, socioeconomic deprivation, the secular year of index AF diagnosis, ethnicity, smoking, CHA_2_DS_2_-VASc score, and the 18 aforementioned comorbidities at baseline. We performed a subsequent analysis adding the Charlson comorbidity index to the previous model to further account for potential frailty of the population. The primary causes of death during follow-up were compared by ICD chapters among treatment groups, and five frequent ICD chapters were chosen for that purpose.

We performed the analyses in the secured Data Safe Haven, meeting the data safety and information governance requirements by University College London, NHS Digital, and ONS. Analyses were performed in Statistical Analysis System (version 9.4) and R (version 3.6.1). The funders did not have any role in study design, data collection, data analysis, interpretation, and writing of the report.

## Results

We identified 199 433 individuals (mean age at diagnosis 75.7 ± 12.7 years; 50.2% women) with new-onset AF diagnosis between 1 January 1998 and 31 May 2016 in the study (see Supplementary material online, *Figure S1*). The median follow-up among AF patients after the initial diagnosis was 2.7 (0.7–6.0) years. A small portion of new-onset AF patients received rhythm control treatment prior to or at study entry (see Supplementary material online, *Table S4*).

During follow-up, 28 497 (14.3%) of new-onset AF patients had initiated either anti-arrhythmic medication (*n* = 27 651) or invasive treatment (*n* = 2997). The mean duration between new-onset AF diagnosis and initial rhythm control treatment differed from 1.2 years for amiodarone use and 3.6 years for pulmonary vein isolation (see Supplementary material online, *Table S4*).

Comparing new-onset AF patients receiving rhythm control treatment to those who did not, the proportion of women was lower, while the prevalence of comorbidities was lower (*Table 1*). The presence of ventricular or supraventricular tachycardia at study entry was higher in patients receiving rhythm control. Comparing the a priori subgroups among the 28 497 AF patients receiving rhythm control treatment or invasive rate control, we found more men in the younger subgroup (68.5%) and more women in the advanced age group (46.9%) (*Table 2*). Compared with men, more female patients receiving rhythm control treatment had a history of valvular diseases or tachycardia at the time of their new-onset AF diagnosis. Patients who received rhythm control treatment living in areas with a higher socioeconomic deprivation had a greater prevalence of comorbidity than those living in more wealthy areas (*Table 2*).

**Table 1:** Baseline characteristics of individuals with AF with and without rhythm control treatment

Baseline characteristics	Rhythm control treatment, *n* (%)	No rhythm control treatment, *n* (%)	Baseline characteristics	Rhythm control treatment, *n* (%)	No rhythm control treatment, *n* (%)
*n*	28 497	196 676	*n*	28 497	196 676
Age	62.2 ± 13.7	69.0 ± 13.0	Asthma	3559 (12.5%)	19 172 (11.9%)
Women	12 191 (42.8%)	83 343 (51.7%)	COPD	2174 (7.6%)	17 507 (10.9%)
Least deprived quintile	5555 (19.5%)	26 326 (16.3%)	Hyperthyroidism	409 (1.4%)	3247 (2%)
Most deprived quintile	5787 (20.3%)	36 583 (22.7%)	Cancer	4165 (14.6%)	35 615 (22.1%)
Smoking	11 527 (40.4%)	67 175 (41.7%)	CKD	2427 (8.5%)	27 529 (17.1%)
CHA_2_DS_2_-VASc score (≥1 for men and ≥2 for women)	23 193 (81.4%)	150 284 (93.3%)	COPD	2174 (7.6%)	17 507 (10.9%)
Hypertension	12 134 (42.6%)	79 248 (49.2%)	Dementia	211 (0.7%)	7380 (4.6%)
DM Type 2	3202 (11.2%)	22 870 (14.2%)	Direct oral anticoagulants	235 (0.8%)	2485 (1.5%)
Valvular heart disease	2639 (9.3%)	9544 (5.9%)	Warfarin	2151 (7.5%)	14 206 (8.8%)
Mitral valve disease	1130 (4%)	4607 (2.9%)	Antiplatelets	11 609 (40.7%)	72 634 (45.1%)
Stable angina	4635 (16.3%)	22 591 (14%)	Anti-arrhythmic agents Class I and III	698 (2.4%)	3550 (2.2%)
Ischaemic heart disease	5012 (17.6%)	23 307 (14.5%)	Beta-blockers	10 679 (37.5%)	56 128 (34.8%)
MI	3955 (13.9%)	18 869 (11.7%)	Calcium channel blockers	8615 (30.2%)	55 636 (34.5%)
Heart failure	3104 (10.9%)	22 548 (14%)	Cardiac glycosides	2046 (7.2%)	16 217 (10.1%)
SVT	1150 (4%)	2704 (1.7%)	Diuretics	11 564 (40.6%)	81 978 (50.9%)
VT	1351 (4.7%)	4320 (2.7%)	Statins	8521 (29.9%)	49 625 (30.8%)
Stroke	1620 (5.7%)	19 671 (12.2%)	NSAID	15 732 (55.2%)	86 468 (53.7%)
TIA	1469 (5.2%)	12 458 (7.7%)			
PE	615 (2.2%)	4713 (2.9%)			
DVT	864 (3%)	7171 (4.4%)			
PAD	1641 (5.8%)	12 286 (7.6%)			

CKD, chronic kidney disease; COPD, chronic obstructive pulmonary disease; DM, diabetes mellitus; DVT, deep vein thrombosis; MI, myocardial infarction; NSAID, non-steroidal anti-inflammatory drugs; PE, pulmonary embolism; PAD, peripheral artery disease; SVT, supraventricular tachycardia; TIA, transient ischaemic attack; VT, ventricular tachycardia.

**Table 2:** Baseline characteristics of individuals with AF with rhythm control treatment by sex, age, and socioeconomic categories

	<70 years	≥ 70 years	Male	Female	Low IMD	High IMD
*n*	13 633	14 864	16 306	12 191	5555	5787
Women	4291 (31.5%)	7990 (53.1%)	–	–	2417 (43.5%)	2452 (42.4%)
Least deprived quintile	2531 (18.6%)	3024 (20.3%)	3138 (19.2%)	2417 (19.8%)	–	–
Most deprived quintile	2851 (20.9%)	2936 (19.8%)	3335 (20.5%)	2452 (20.1%)	–	–
Smoking	5744 (42.1%)	5783 (38.9%)	7717 (47.3%)	3810 (31.3%)	2024 (36.4%)	2639 (34.6%)
CHA_2_DS_2_-VASc score (≥1 for men and ≥2 for women)	8329 (61.1%)	14 864 (100%)	12 528 (76.8%)	10 665 (87.5%)	4512 (81.2%)	4746 (82%)
Hypertension	4621 (33.9%)	7513 (50.5%)	6320 (38.8%)	5814 (47.7%)	2315 (41.7%)	2520 (43.5%)
DM Type 2	1355 (9.9%)	1847 (12.4%)	2007 (12.3%)	1195 (9.8%)	521 (9.4%)	716 (12.4%)
Valvular heart disease	1089 (8%)	1550 (10.4%)	1456 (8.9%)	1183 (9.7%)	532 (9.6%)	568 (9.8%)
Mitral valve disease	548 (4%)	582 (3.9%)	569 (3.5%)	561 (4.6%)	254 (4.6%)	228 (3.9%)
Stable angina	1498 (11%)	3137 (21.1%)	2854 (17.5%)	1781 (14.6%)	792 (14.3%)	1144 (19.8%)
Ischaemic heart disease	1832 (13.4%)	3180 (21.4%)	3396 (20.8%)	1616 (13.3%)	854 (15.4%)	1148 (19.8%)
MI	1431 (10.5%)	2524 (17%)	2805 (17.2%)	1150 (9.4%)	658 (11.8%)	918 (15.9%)
Heart failure	981 (7.2%)	2123 (14.3%)	1791 (11%)	1313 (10.8%)	506 (9.1%)	765 (13.2%)
SVT	581 (4.3%)	569 (3.8%)	589 (3.6%)	561 (4.6%)	233 (4.2%)	230 (4%)
VT	654 (4.8%)	697 (4.7%)	700 (4.3%)	651 (5.3%)	289 (5.2%)	264 (4.6%)
Stroke	476 (3.5%)	1144 (7.7%)	865 (5.3%)	754 (6.2%)	308 (5.5%)	322 (5.6%)
TIA	390 (2.9%)	1079 (7.3%)	782 (4.8%)	687 (5.6%)	296 (5.3%)	308 (5.3%)
PE	222 (1.6%)	393 (2.6%)	310 (1.9%)	305 (2.5%)	105 (1.9%)	125 (2.2%)
DVT	329 (2.4%)	535 (3.6%)	459 (2.8%)	405 (3.3%)	164 (3%)	176 (3%)
PAD	493 (3.6%)	1148 (7.7%)	991 (6.1%)	650 (5.3%_	271 (4.9%)	376 (6.5%)
Asthma	1700 (12.5%)	1859 (12.5%)	1827 (11.2%)	1732 (14.2%)	711 (12.9%)	777 (13.4%)
COPD	710 (5.2%)	1464 (9.8%)	1337 (8.2%)	837 (6.9%)	304 (5.5%)	601 (10.4%)
Hyperthyroidism	167 (1.2%)	242 (1.6%)	103 (0.6%)	306 (2.5%)	69 (1.2%)	89 (1.5%)
Cancer	1183 (8.7%)	2982 (20.1%)	2251 (13.8%)	1914 (15.7%)	856 (15.4%)	787 (13.6%)
CKD	706 (5.2%)	1721 (11.6%)	1221 (7.5%)	1206 (9.9%)	425 (7.7%)	592 (10.2%)
Dementia	11 (0.1%)	200 (1.3%)	77 (0.5%)	134 (1.1%)	39 (0.7%)	43 (0.7%)
Direct oral anticoagulants	109 (0.8%)	126 (0.8%)	125 (0.8%)	110 (0.9%)	43 (0.8%)	51 (0.9%)
Warfarin	1000 (7.3%)	1151 (7.7%)	1326 (8.1%)	825 (6.8%)	407 (7.3%)	434 (7.5%)
Antiplatelets	4191 (30.7%)	7418 (49.9%)	6827 (41.9%)	4782 (39.2%)	2215 (39.9%)	2543 (43.9%)
Anti-arrhythmic agents Class I and III	271 (2%)	427 (2.9%)	308 (1.9%)	390 (3.2%)	131 (2.4%)	170 (2.9%)
Beta-blockers	4757 (34.9%)	5922 (39.8%)	591 (36.3%)	4758 (39%)	2094 (37.7%)	2281 (39.4%)
Calcium channel blockers	3125 (22.9%)	5490 (36.9%)	4739 (29.1%)	3876 (31.8%)	1573 (28.3%)	1965 (34%)
Cardiac glycosides	723 (5.3%)	1323 (8.9%)	1054 (6.5%)	992 (8.1%)	415 (7.5%)	417 (7.2%)
Diuretics	3881 (28.5%)	7683 (51.7%)	5543 (34%)	6021 (49.4%)	2182 (39.3%)	2550 (44.1%)
Statins	3496 (25.6%)	5025 (33.8%)	5368 (32.9%)	3153 (25.9%)	1600 (28.8%)	1893 (32.7%)
NSAID	7197 (52.8%)	8535 (57.4%)	8709 (53.4%)	7023 (57.6%)	3100 (55.8%)	3352 (57.9%)

CKD, chronic kidney disease; COPD, chronic obstructive pulmonary disease; DM, diabetes mellitus; DVT, deep vein thrombosis; IMD, indices of multiple deprivation; MI, myocardial infarction; NSAID, non-steroidal anti-inflammatory drugs; PAD, peripheral artery disease; PE, pulmonary embolism; TIA, transient ischaemic attack; SVT, supraventricular tachycardia; VT, ventricular tachycardia.

At baseline, fewer (8.0%) patients in the rhythm control group were treated with oral anticoagulants or warfarin than comparisons (9.7%), whereas the proportion increased during follow-up (60.2% in the rhythm control group and 33.4% in comparisons).

The most used anti-arrhythmic drug was amiodarone (62%), and about one-third received treatment with sotalol (31%). Flecainide and propafenone were used in 15 and 1%, respectively (*Table 3*). Pulmonary vein isolation and ablation for flutter were used in only 5% of patients receiving rhythm control. The use of ablation was four times higher in the younger than older subgroups (17.8 vs. 3.9%), and pulmonary vein isolation only accounted for 1% of rhythm control approaches among individuals who were 70 years and older. The proportion of ablation was also higher among male patients than females, and higher in patients living in wealthier areas than those living in deprived areas.

**Table 3:** Rhythm control or invasive AF treatment by sex, age, and socioeconomic categories

Rhythm control treatment methods	Strata in rhythm control groups						
	All	<70 years	≥ 70 years	Male	Female	Low IMD	High IMD
*N*	28 497	13 633	14 864	16 306	12 191	5555	5787
Amiodarone	17 597 (61.8%)	7271 (53.3%)	10 326 (69.5%)	10 558 (64.7%)	7039 (57.7%)	3236 (58.3%)	3801 (65.7%)
Flecainide	4398 (15.4%)	3406 (25%)	992 (6.7%)	2437 (14.9%)	1961 (16.1%)	1035 (18.6%)	667 (11.5%)
Propafenone	320 (1.1%)	235 (1.7%)	85 (0.6%)	170 (1%)	150 (1.2%)	117 (2.1%)	55 (1%)
Sotalol	8895 (31.2%)	4469 (32.8%)	4426 (29.8%)	4484 (27.5%)	4411 (36.2%)	1820 (32.8%)	1668 (28.8%)
Any ablation	2997 (10.5%)	2414 (17.8%)	583 (3.9%)	2122 (13.1%)	875 (7.2%)	659 (11.9%)	502 (8.7%)
PVI	1342 (4.7%)	1191 (8.7%)	151 (1%)	946 (5.8%)	396 (3.2%)	306 (5.5%)	221 (3.8%)
Atrial flutter ablation	1364 (4.8%)	1061 (7.8%)	303 (2%)	1036 (6.4%)	328 (2.7%)	302 (5.4%)	226 (3.9%)
PVI or flutter ablation	2306 (8.1%)	1897 (13.9%)	409 (2.8%)	1677 (10.3%)	629 (5.2%)	509 (9.2%)	381 (6.6%)

PVI, pulmonary vein isolation.

**Figure 1:**
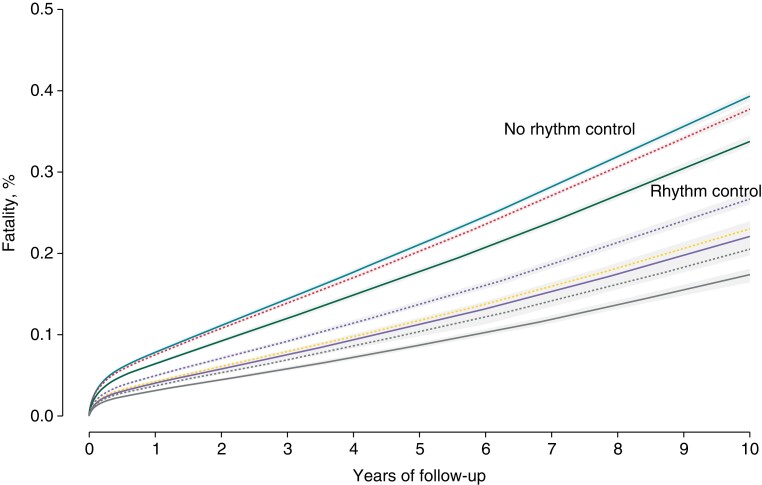
Age- and sex-adjusted Kaplan–Meier analyses for cumulative incidence of study outcomes by AF treatment groups. From higher fatality to lower fatality: sea blue, no rhythm control; dashed red, amiodarone use; green, rhythm control; dashed purple, sotalol use; dashed yellow, propafenone use; purple, flutter-only ablation; dashed gray, flecainide; gray, pulmonary vein isolation.

Adjusted Kaplan–Meier analyses of all-cause mortality of new-onset AF patients showed that, after accounting for age, sex, and varying therapy starting times, patients receiving rhythm control treatment had significantly lower mortality than patients not receiving rhythm control treatment (*P* < 0.001, *Figure 1*). The age- and sex-adjusted analyses showed that different rhythm control treatments were all associated with better survival. Crude analysis of patients receiving anticoagulants during follow-up showed that the mortality, assessed as proportion of deaths, was lower in the rhythm control group (27.6 vs. 33.4%; *P* < 0.001).

The most pronounced risk reduction was observed for pulmonary vein isolation (adjusted HR, pulmonary vein isolation compared with no rhythm control: 0.28, 95% confidence interval (CI) 0.21–0.36), followed by flecainide, the use of propafenone, atrial flutter-only ablation, sotalol use, and amiodarone use (*Figure 1*).

### Multivariate analysis

**Figure 2:**
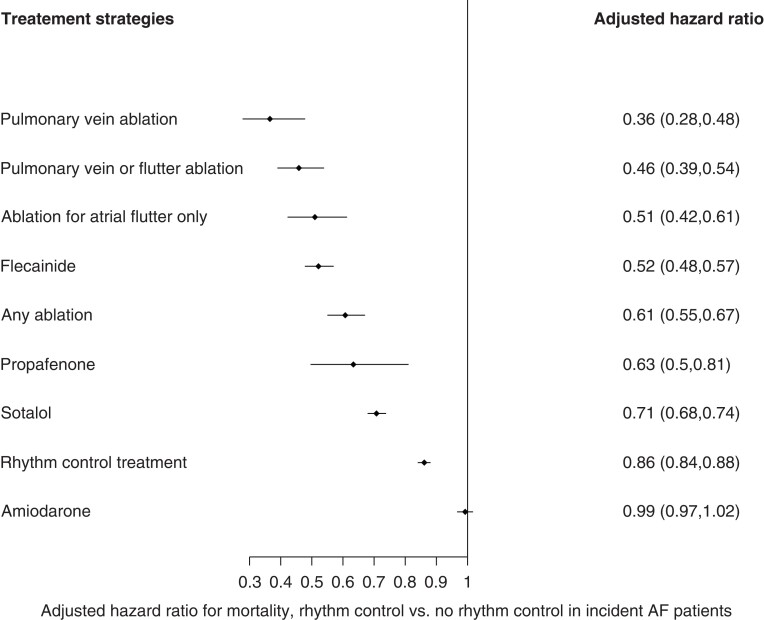
Multivariate-adjusted Cox regression analyses for mortality risk by rhythm control or invasive treatment strategies.

On multivariate-adjusted Cox regression analyses, we observed that rhythm control treatment reduced mortality among new-onset AF patients by 14% (adjusted HR = 0.86, 95% CI 0.84–0.88) (*Figure 2*). Pulmonary vein isolation was associated with a two-third mortality reduction compared with no rhythm control (adjusted HR = 0.36, 95% CI 0.28–0.48). Flecainide use and flutter-only ablation were associated with a 50% mortality risk reduction (HR = 0.52, 95% CI 0.48–0.57, and HR = 0.51, 95% CI 0.42–0.61, respectively). Propafenone use and sotalol use were associated with about a third reduction in mortality (HR = 0.63, 95% CI 0.50–0.81, and HR = 0.71, 95% CI 0.68–0.74, respectively). The use of amiodarone had no significant impact on mortality when compared with no rhythm control therapy (HR = 0.99, 95% CI 0.97–1.02).

An additional analysis, adding further adjustment with the Charlson comorbidity index showed similar mortality reduction effect with rhythm control (HR = 0.87, 95% CI 0.85–0.89).

### Subgroup analyses

Analyses by a priori subgroups showed that rhythm control treatment was associated with similar mortality risk for men (HR = 0.68, 95% CI 0.66–0.70) and women (HR = 0.71, 95% CI 0.69–0.73), and new-onset AF patients with a history of heart failure (HR = 0.73, 95% CI 0.70–0.77) or without heart failure history (HR = 0.69, 95% CI 0.67, 0.70) or in patients older than 70 years (HR = 0.76, 95% CI 0.75–0.78) and in those younger than 70 years of age at study entry (HR = 0.78, 95% CI 0.74–0.82).

In a detailed Investigation of rhythm control methods in patient subgroups, there was a potential survival benefit of the use of amiodarone among all subgroups, except for a null effect in individuals with AF younger than 70 years at baseline (HR = 1.04, 95% CI 0.98–1.09) (see Supplementary material online, *Figure S2*).

A sub-analysis of two time periods (before 2006 and from 2006 onwards), showed a growth in the utilization of catheter ablation and flecainide and drop in the use of amiodarone and sotalol (see Supplementary material online, *Table S5*). Multivariate cox regression analyses for the two time periods showed a more pronounced mortality reduction with rhythm control in recent years (see Supplementary material online, *Table S6*).

**Figure 3:**
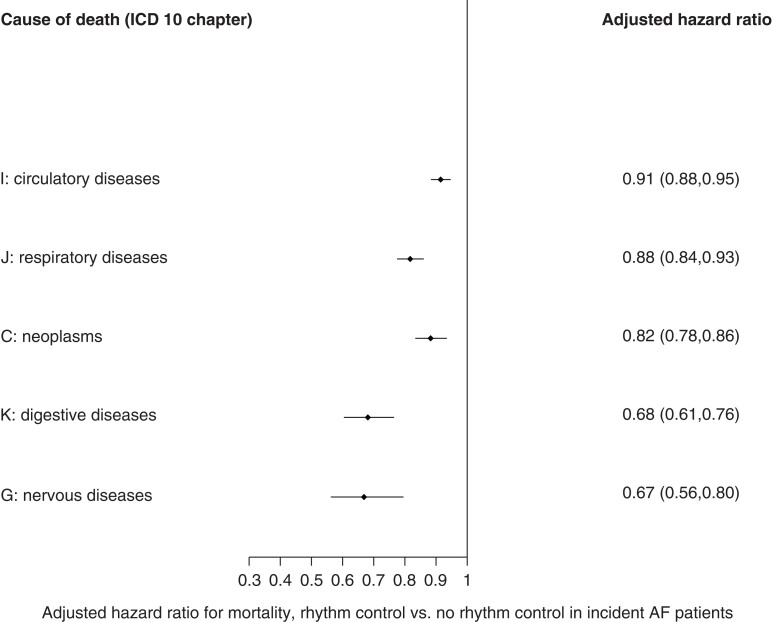
Cause of death by treatment methods.

Five-year mortality varied across treatment options (see Supplementary material online, *Table S7*). The leading causes of death in the study population were diseases of the circulatory system, neoplasms, diseases of the respiratory system, and diseases of the digestive system (see Supplementary material online, *Table S8*). Individuals with AF receiving rhythm control treatment had a lower proportion of deaths due to circulatory system, neoplasms, diseases of the respiratory system, and from diseases of the digestive or nervous systems than individuals without rhythm control treatment (*Figure 3*).

## Discussion

The study provides evidence for our understanding of the utilization and outcome of rhythm control options for managing AF in the routine care setting in the UK. We reported that while favourable survival was observed in almost all rhythm control methods, there may be a difference in survival among the different rhythm control treatment strategies. The effect of rhythm control on survival did not differ by age, sex, or history of heart failure, whereas we report a null effect in the use of amiodarone among AF patients younger than 70 years at their index diagnosis.

Our study shows that among different rhythm control strategies, pulmonary vein isolation seems to show the most pronounced survival benefit. Flecainide seems to be the anti-arrhythmic agent with the best survival benefit easing away some concerns regarding class IC agents and increased mortality, which come from trials where these were used to treat ventricular ectopy in patients with ischaemic heart disease.^15^ The survival benefit seems to be lower with sotalol. Interestingly, a previous Cochrane systematic review showed increased mortality with sotalol when used to maintain sinus rhythm following cardioversion,^16^ which we could not confirm in our data. On the other hand, amiodarone does not increase mortality but has no benefit over non-rhythm control. Our findings corroborate a previous observation in a Danish Nationwide study which showed that flecainide was associated with a significant 62% reduction in all-cause mortality (HR = 0.38, 95% CI 0.32–0.44), propafenone and sotalol associated with slightly less pronounced benefit (HR = 0.65, 95% CI 0.58–0.71, and HR = 0.65, 95% CI 0.63–0.67, respectively), and results were neutral or showing only very mild benefit for amiodarone (HR = 0.94, 95% CI 0.89–1.00).^17^ With regard to survival benefit for catheter ablation, analysis of a Korean National data set has shown a similar magnitude of effect (HR = 0.41, 95% CI 0.36–0.47) to the one we observed.^18^

Our cause-of-death analyses showed that deaths due to disease of the circulatory system were less frequent in patients under rhythm control. This suggests that rhythm control, and consequently a lower AF burden, may have an important prognostic impact across the spectrum of cardiovascular disease. This finding appears to be of importance as cardiovascular disease is the leading cause of death in the UK and Worldwide.^19^

### Practice implications and future research

RACE and AFFIRM trials failed to show a survival benefit of rhythm control in AF patients.^20,21^ However, in these trials, patients in the rhythm control group more frequently stopped anticoagulants, with a few developing strokes during follow-up. In our study, the proportion of anticoagulant use increased during follow-up, more in the rhythm control group than in comparisons, and may partly explain the observed survival benefits in patients receiving rhythm control strategy. A previous real-world study of pulmonary vein isolation in the heart failure population shows survival benefit of catheter ablation.^22^ In our study, where follow-up duration was much longer than the existing trials, we observed that the survival benefit of rhythm control was present both for patients with and without a history of heart failure. Future trials on the impact of catheter ablation of AF in the non-heart failure population may be required to confirm this observation.

Our study is the largest comparison of different rhythm control strategies vs. no rhythm control. The EAST-AFNET4 study investigated 2789 AF patients and showed survival benefits of rhythm control. However, the number of AF patients did not enable a direct comparison of the different rhythm control strategies.^7^ A previous analyses of primary care data from Germany suggested that some anti-arrhythmic agents (i.e. dronedarone) could be safer and associated with a lower rate of stroke or myocardial infarction.^23^ In this study, dronedarone was compared with all other anti-arrhythmic agents that formed a control group. It would be of interest to assess the rate of the aforementioned cardiovascular events for each one of them (e.g. sotalol, flecainide, propafenone, and amiodarone). An analysis of the Swedish patient register assessed the impact of catheter ablation for AF on mortality and stroke but provided no information on the impact of the different anti-arrhythmic agents on the same outcomes.^24^ Similarly, other studies have reported on findings for anti-arrhythmic drugs^17^ or catheter ablation^18^ but not for all strategies combined.

Our study is the first real-world analysis of the impact of the different rhythm control strategies on mortality, showing a survival benefit with catheter ablation and most anti-arrhythmic agents, with a warning sign for amiodarone in individuals aged <70 years. However, our observational study design, methodology, and population (real-world data) are different from a highly selected population and randomized design of the abovementioned trials.^7,20,21^

### Limitations

This study was performed in an EHR UK data set having all its inherent limitations. A further randomized controlled trial is warranted to confirm our findings. Secondly, rhythm control strategies were utilized only in a minor proportion of AF patients, suggesting possible selection bias. This is likely to be related to the weak indication in the guidelines in the 2000s: the 2006 jointly European and North American guidelines recommended rhythm control only to patients with disabling symptoms;^25^ subsequently, the 2010 European guideline^26^ still suggests that rhythm control should be reserved for selected patients (‘Rate control is needed for most patients with AF unless the heart rate during AF is naturally slow. Rhythm control may be added to rate control if the patient is symptomatic despite adequate rate control or if a rhythm control strategy is selected because of factors such as the degree of symptoms, younger age, or higher activity levels’). Thirdly, this data set did not contain enough data on dronedarone to allow meaningful analyses, but this may reflect the low utilization of this drug in the UK. Fourthly, ICD-10 coding did not allow us to discriminate atrial flutter and AF patients in this data set, and therefore, strong inferences on the impact of atrial flutter ablation on survival cannot be made. Fifthly, no information on left atrial size was available in our data set. Still, the impact of left atrial dilation on survival remains to be proven in the AF population.^27^ Despite adjusting for 25 variables in our model, our time-to-event curves diverge earlier than in the trials, suggesting the presence of some residual confounding. Finally, there is the risk of unmeasured risk factors or comorbidities. We managed this limitation by including in our analyses the adjustment of 25 key risk factors supported by the previous literature relating to AF and clinical outcome.

## Conclusion

We report the favourable survival associated with rhythm control treatment among individuals with new-onset AF. Among different rhythm control strategies, pulmonary vein isolation seems to show the most pronounced survival benefit. The effect of rhythm control on survival does not differ by age, sex, nor history of heart failure.

## Supplementary Material

euac155_Supplementary_Data

## Data Availability

Data were provided by the Medicines & Healthcare products Regulatory Agency upon approval of Protocol 17_205R by the Independent Scientific Advisory Committee.
