# Supplementary material for: Impact of anti-arrhythmic drugs and catheter ablation on the survival of patients with atrial fibrillation: a population study based on 199 433 new-onset atrial fibrillation patients in the UK
Source: Europace. 2022 Sep 15;25(2):351–9. doi: 10.1093/europace/euac155 (PMC10103560; doi:10.1093/europace/euac155)
Supplement: euac155_Supplementary_Data [file euac155_supplementary_data.pdf]

Supplemental File (visible in PDF)
Click here to access/download;Supplemental File (visible in PDF);Supplementary appendix 7072022.docx

Supplementary appendix of the study

Different Rhythm Control Strategies on the Outcomes of Atrial Fibrillation in the United Kingdom

Sheng-Chia Chung, Alvina Lai, Gregory Lip, Pier D Lambiase, Rui Providencia

Table S1: The definition of antiarrhythmic drugs in health records

productname	drugsubstance	formulation	route	bnfchapter	Anti-arrhythmic class
amiodarone tablets 200mg	amiodarone hydrochloride	tablets	Oral	Supraventricular & ventricular arrhythmias	amiodarone
amiodarone tablets 100mg	amiodarone hydrochloride	tablets	Oral	Supraventricular & ventricular arrhythmias	amiodarone
sotalol tablets 40mg	sotalol hydrochloride	tablets	Oral	Supraventricular & ventricular arrhythmias/Beta-adrenoceptor blocking drugs	sotalol
flecainide acetate tablets 50mg	flecainide acetate	tablets	Oral	Supraventricular & ventricular arrhythmias	flecainide
flecainide acetate tablets 100mg	flecainide acetate	tablets	Oral	Supraventricular & ventricular arrhythmias	flecainide
sotalol tablets 80mg	sotalol hydrochloride	tablets	Oral	Supraventricular & ventricular arrhythmias/Beta-adrenoceptor blocking drugs	sotalol
ARYTHMOL tablets 150mg [ABBOTT]	propafenone hydrochloride	tablets	Oral	Supraventricular & ventricular arrhythmias	proprafenone
sotalol with hydrochlorothiazide tablets 160mg + 25mg	hydrochlorothiazide/sotalol hydrochloride	tablets	Oral	Beta-adrenoceptor blocking drugs	sotalol
SOTACOR tablets 80mg [BRISTOL]	sotalol hydrochloride	tablets	Oral	Supraventricular & ventricular arrhythmias/Beta-adrenoceptor blocking drugs	sotalol
propafenone hydrochloride tablets 150mg	propafenone hydrochloride	tablets	Oral	Supraventricular & ventricular arrhythmias	proprafenone
BETA-CARDONE tablets 40mg [UCB]	sotalol hydrochloride	tablets	Oral	Supraventricular & ventricular arrhythmias/Beta-adrenoceptor blocking drugs	sotalol
BETA-CARDONE tablets 80mg [UCB]	sotalol hydrochloride	tablets	Oral	Supraventricular & ventricular arrhythmias/Beta-adrenoceptor blocking drugs	sotalol
sotalol with hydrochlorothiazide tablets 80mg + 12.5mg	hydrochlorothiazide/sotalol hydrochloride	tablets	Oral	Beta-adrenoceptor blocking drugs	sotalol
CORDARONE X tablets 200mg [SANOFI S]	amiodarone hydrochloride	tablets	Oral	Supraventricular & ventricular arrhythmias	amiodarone
propafenone hydrochloride tablets 300mg	propafenone hydrochloride	tablets	Oral	Supraventricular & ventricular arrhythmias	proprafenone
sotalol tablets 160mg	sotalol hydrochloride	tablets	Oral	Supraventricular & ventricular arrhythmias/Beta-adrenoceptor blocking drugs	sotalol

TAMBOCOR tablets 100mg [MEDA]	flecainide acetate	tablets	Oral	Supraventricular & ventricular arrhythmias	flecainide
SOTACOR tablets 160mg [BRISTOL]	sotalol hydrochloride	tablets	Oral	Supraventricular & ventricular arrhythmias/Beta-adrenoceptor blocking drugs	sotalol
CORDARONE X tablets 100mg [SANOFI S]	amiodarone hydrochloride	tablets	Oral	Supraventricular & ventricular arrhythmias	amiodarone
SOTAZIDE tablets [BRISTOL]	hydrochlorothiazide/sotalol hydrochloride	tablets	Oral	Beta-adrenoceptor blocking drugs	sotalol
sotalol tablets 200mg	sotalol hydrochloride	tablets	Oral	Supraventricular & ventricular arrhythmias/Beta-adrenoceptor blocking drugs	sotalol
BETA-CARDONE tablets 200mg [UCB]	sotalol hydrochloride	tablets	Oral	Supraventricular & ventricular arrhythmias/Beta-adrenoceptor blocking drugs	sotalol
TAMBOCOR tablets 50mg [MEDA]	flecainide acetate	tablets	Oral	Supraventricular & ventricular arrhythmias	flecainide
flecainide acetate oral suspension 25mg/5ml	flecainide acetate	oral suspension	Oral	Supraventricular & ventricular arrhythmias/Unlicensed medicinal product (specials)	flecainide
TOLERZIDE tablets [BMS]	hydrochlorothiazide/sotalol hydrochloride	tablets	Oral	Beta-adrenoceptor blocking drugs	sotalol
ARYTHMOL tablets 300mg [ABBOTT]	propafenone hydrochloride	tablets	Oral	Supraventricular & ventricular arrhythmias	proprafenone
AMIDOX tablets 200mg [BERK]	amiodarone hydrochloride	tablets	Oral	Supraventricular & ventricular arrhythmias	amiodarone
AMIODARONE tablets 200mg [SANDOZ]	amiodarone hydrochloride	tablets	Oral	Supraventricular & ventricular arrhythmias	amiodarone
FLECAINIDE ACETATE tablets 100mg [WINTHROP]	flecainide acetate	tablets	Oral	Supraventricular & ventricular arrhythmias	flecainide
FLECAINIDE ACETATE tablets 50mg [HILLCROSS]	flecainide acetate	tablets	Oral	Supraventricular & ventricular arrhythmias	flecainide
AMIODARONE tablets 100mg [HILLCROSS]	amiodarone hydrochloride	tablets	Oral	Supraventricular & ventricular arrhythmias	amiodarone
FLECAINIDE ACETATE tablets 50mg [GEN (UK)]	flecainide acetate	tablets	Oral	Supraventricular & ventricular arrhythmias	flecainide
AMIODARONE tablets 200mg [HILLCROSS]	amiodarone hydrochloride	tablets	Oral	Supraventricular & ventricular arrhythmias	amiodarone

AMYBEN tablets 100mg [LEXON]	amiodarone hydrochloride	tablets	Oral	Supraventricular & ventricular arrhythmias	amiodarone
SOTALOL tablets 40mg [HILLCROSS]	sotalol hydrochloride	tablets	Oral	Supraventricular & ventricular arrhythmias/Beta-adrenoceptor blocking drugs	sotalol
AMIODARONE tablets 200mg [GEN (UK)]	amiodarone hydrochloride	tablets	Oral	Supraventricular & ventricular arrhythmias	amiodarone
SOTALOL tablets 80mg [GEN (UK)]	sotalol hydrochloride	tablets	Oral	Supraventricular & ventricular arrhythmias/Beta-adrenoceptor blocking drugs	sotalol
FLECAINIDE ACETATE tablets 100mg [GEN (UK)]	flecainide acetate	tablets	Oral	Supraventricular & ventricular arrhythmias	flecainide
SOTALOL tablets 40mg [TEVA]	sotalol hydrochloride	tablets	Oral	Supraventricular & ventricular arrhythmias/Beta-adrenoceptor blocking drugs	sotalol
SOTALOL tablets 40mg [TILLOMED]	sotalol hydrochloride	tablets	Oral	Supraventricular & ventricular arrhythmias/Beta-adrenoceptor blocking drugs	sotalol
SOTALOL tablets 80mg [SANDOZ]	sotalol hydrochloride	tablets	Oral	Supraventricular & ventricular arrhythmias/Beta-adrenoceptor blocking drugs	sotalol
AMIODARONE tablets 200mg [WINTHROP]	amiodarone hydrochloride	tablets	Oral	Supraventricular & ventricular arrhythmias	amiodarone
FLECAINIDE ACETATE tablets 50mg [TEVA]	flecainide acetate	tablets	Oral	Supraventricular & ventricular arrhythmias	flecainide
AMIODARONE tablets 200mg [TEVA]	amiodarone hydrochloride	tablets	Oral	Supraventricular & ventricular arrhythmias	amiodarone
amiodarone oral suspension 25mg/5ml	amiodarone hydrochloride	oral suspensi on	Oral	Supraventricular & ventricular arrhythmias/Unlicensed medicinal product (specials)	amiodarone
flecainide acetate modified release capsules 200mg	flecainide acetate	modified release capsules	Oral	Supraventricular & ventricular arrhythmias	flecainide
sotalol oral suspension 25mg/5ml	sotalol hydrochloride	oral suspensi on	Oral	Supraventricular & ventricular arrhythmias/Beta-adrenoceptor blocking drugs/Unlicensed medicinal product (specials)	sotalol
sotalol injection 40mg/4ml	sotalol hydrochloride	injection	Unkn own	Supraventricular & ventricular arrhythmias/Beta-adrenoceptor blocking drugs	sotalol
SOTALOL tablets 80mg [HILLCROSS]	sotalol hydrochloride	tablets	Oral	Supraventricular & ventricular arrhythmias/Beta-adrenoceptor blocking drugs	same comment on iv drugs.
TAMBOCOR XL capsules 200mg [MEDA]	flecainide acetate	capsules	Oral	Supraventricular & ventricular arrhythmias	flecainide

AMIODARONE tablets 200mg [IVAX]	amiodarone hydrochloride	tablets	Oral	Supraventricular & ventricular arrhythmias	amiodarone
AMIODARONE tablets 100mg [IVAX]	amiodarone hydrochloride	tablets	Oral	Supraventricular & ventricular arrhythmias	amiodarone
FLECAINIDE ACETATE tablets 100mg [ACTAVIS]	flecainide acetate	tablets	Oral	Supraventricular & ventricular arrhythmias	flecainide

Table S2: The definition of ablation in health records

code	term	dictionary	medcode
**Pulmonary vein ablation**			
K575	Percutaneous transluminal ablation of atrial wall NEC	opcs	NA
K621	Percutaneous transluminal ablation of pulmonary vein to left atrium conducting system	opcs	NA
7934500	Percutaneous transluminal ablation of atrial wall	read	87338
7934800	Percutaneous transluminal ablation of atrial wall NEC	read	89357
**Ablation for atrial flutter only**			
K622	Percutaneous transluminal ablation of atrial wall for atrial flutter	opcs	NA
K623	Percutaneous transluminal ablation of conducting system of heart for atrial flutter NEC	opcs	NA
793M100	Perc transluminal ablation of atrial wall for atrial flutter	read	84152
**Pace and ablate strategy**			
K571	Percutaneous transluminal ablation of atrioventricular node	opcs	NA
K572	Percutaneous transluminal ablation of conducting system of heart NEC	opcs	NA
7930000	Open ablation of atrioventricular node	read	29167
7934000	Percutaneous transluminal ablation of atrioventricular node	read	28933
7934100	Percut transluminal ablation of heart conducting system NEC	read	5746
7934200	Transluminal radiofreq ablation heart conducting system NEC	read	12664

Table S3: Clinical codes used for defining comorbidity in the study .

Exposure and covariates	Definitions
AF	https://www.caliberresearch.org/portal/show/af_hes<br>https://www.caliberresearch.org/portal/show/af_gprd (categories 4,5,6)
Smoking status	https://caliberresearch.org/portal/show/smoking_status_gprd (categories 2,3,4)<br>ICD10: F17
Diabetes	https://www.caliberresearch.org/portal/show/dm_gprd (categories 3,4,6)<br>https://www.caliberresearch.org/portal/show/dm_hes (categories 3,4,6)
Hypertension	https://www.caliberresearch.org/portal/show/ht_gprd (categories 3,4)<br>https://www.caliberresearch.org/portal/show/ht_hes (categories 3,4)
Stable angina	https://www.caliberresearch.org/portal/show/sa_diagnosis_gprd (category 4)<br>https://www.caliberresearch.org/portal/show/angina_hes
Unstable angina	https://www.caliberresearch.org/portal/show/unangina_gprd (category 3)<br>ICD10: I20.0, I24.0, I24.8, I24.9
Myocardial infarction	https://www.caliberresearch.org/portal/show/myo_infarct_gprd (categories 3,4,5)<br>ICD10: I21
stroke	https://www.caliberresearch.org/portal/show/ischaemic_stroke_gprd(category 3)<br>https://www.caliberresearch.org/portal/show/haem_stroke_gprd (categories 3-8)<br>https://www.caliberresearch.org/portal/show/stroke_nos_gprd (category 3)<br>ICD10: I60, I61, I63, I64, I62.0, I62.1, I62.9, G46.3, G46.4, G46.5, G46.6, G46.7,
Dementia	https://www.caliberresearch.org/portal/show/dementia_hes (categories 2-5)<br>https://www.caliberresearch.org/portal/show/dementia_gprd (categories 2-5)
heart failure	https://www.caliberresearch.org/portal/show/hf_gprd (categories 3,4,5,6)<br>https://www.caliberresearch.org/portal/show/hf_hes
Chronic obstructive pulmonary disease	https://www.caliberresearch.org/portal/show/copd_gprd (categories 3,5)<br>https://www.caliberresearch.org/portal/show/copd_hes (categories 3,5)
chronic kidney disease	https://www.caliberresearch.org/portal/show/renal_gprd (categories 3-7)<br>https://www.caliberresearch.org/portal/show/renal_hes (categories 3-7)
cancer	https://www.caliberresearch.org/portal/show/cancer_gprd<br>https://www.caliberresearch.org/portal/show/cancer_hes
Asthma	ICD10 J45, J46 and corresponding Read code
Valvular disease	ICD10: I05. I06. I07, I08, I34, I35, I36, I37 and corresponding Read code.

Figure S1: Flow chart of the study population.

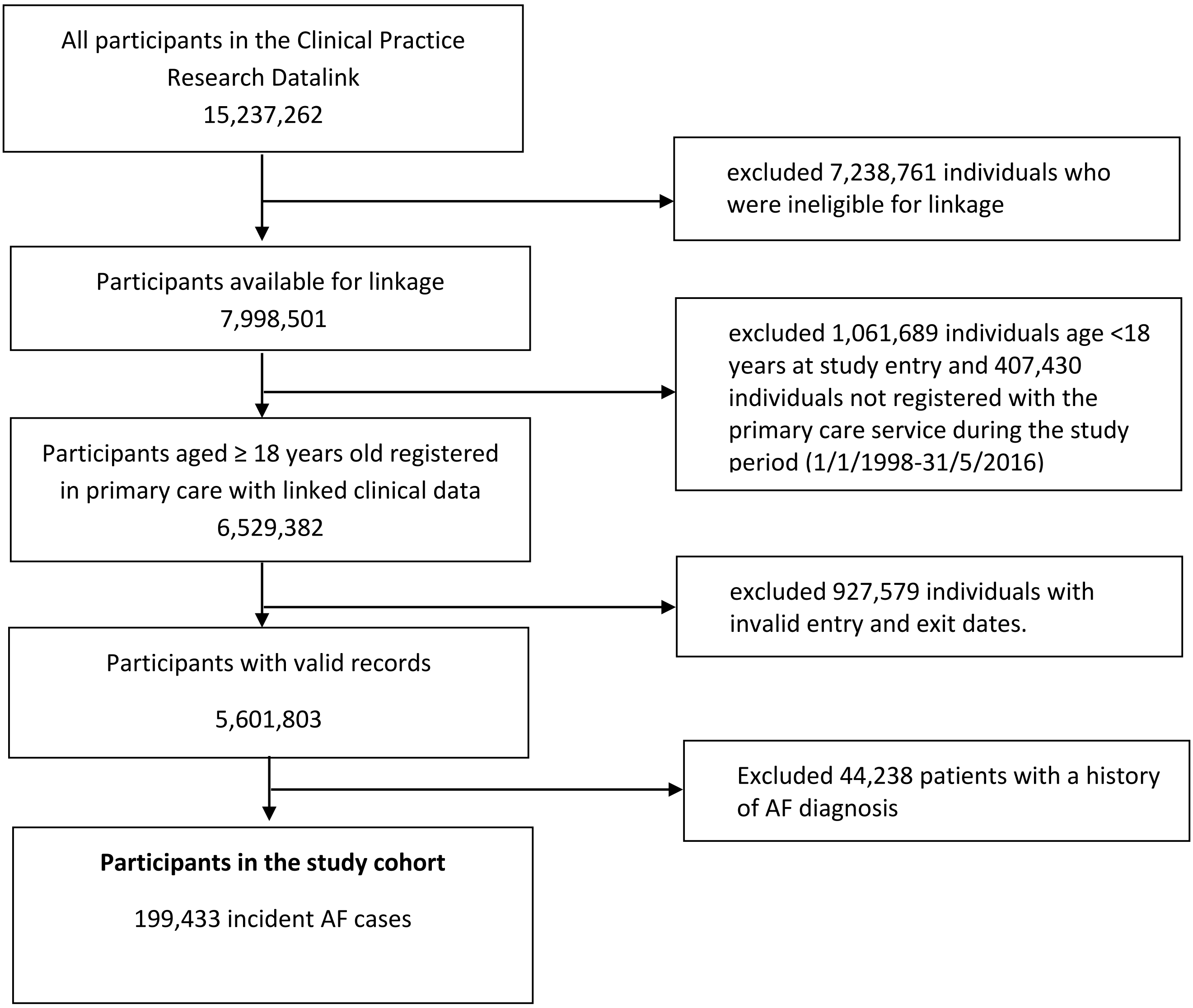

Table S4: The mean duration between incident AF diagnosis and initial rhythm control treatment among AF patients receiving rhythm control treatment.

		Years			
	N (%)	MIN	MAX	MEAN	SD
Age at AF diagnosis in rhythm group	28986	18	99	62.01	13.76
Years between the first rhythm control treatment and AF diagnosis					
Any ablation	4048 (2.98)	0	18	3.517	3.446
Pulmonary vein ablation	1513 (3.31)	0	18	4.296	3.691
Ablation for atrial flutter only	1519 (3.09)	0	18	3.615	3.493
Pace and ablate strategy	2325 (2.98)	0	18	3.425	3.355
Pulmonary vein or flutter ablation	2572 (3.14)	0	18	3.809	3.566
amiodarone	18282 (2.19)	0	18	1.191	2.226
flecainide	4793 (2.69)	0	17	2.025	2.748
proprafenone	390 (2.4)	0	11	2.178	2.384
sotalol	9192 (2.27)	0	16	1.343	2.303

Table S5: A. Utilization of different rhythm control strategies before 2006 and from 2006 onwards.

	AF before 2006	AF after 2006
Amiodarone	9494 (64.1%)	8103 (59.2%)
Flecainide	2039 (13.8%)	2359 (17.2%)
Proprafenone	208 (1.4%)	112 (0.8%)
Sotalol	5237 (35.3%)	3658 (26.7%)
Anyablation	1050 (7.1%)	1947 (14.3%)
PVI	355 (2.4%)	987 (7.2%)
Atrial flutter ablation	319 (2.2%)	1045 (7.6%)
PVI or flutter ablation	565 (3.8%)	1741 (12.7%)

Legend: PVI – pulmonary vein isolation.

Table S6: Impact of rhythm control on mortality before 2006 and from 2006 onwards

	Before 2006	2006 and after
Rhythm control treatment	0.95(0.93,0.98)	0.71(0.68,0.74)
Amiodarone	1.08(1.05,1.11)	0.83(0.79,0.87)
Flecainide	0.59(0.54,0.66)	0.38(0.32,0.45)
Proprafenone	0.71(0.55,0.93)	0.38(0.2,0.72)
Sotalol	0.77(0.73,0.81)	0.59(0.54,0.64)
Any ablation	0.68(0.6,0.77)	0.55(0.48,0.64)
PVI	0.43(0.28,0.67)	0.35(0.25,0.49)
Atrial flutter ablation	0.59(0.42,0.83)	0.52(0.41,0.64)
PVI or flutter ablation	0.55(0.41,0.72)	0.45(0.37,0.54)

Legend: PVI – pulmonary vein isolation.

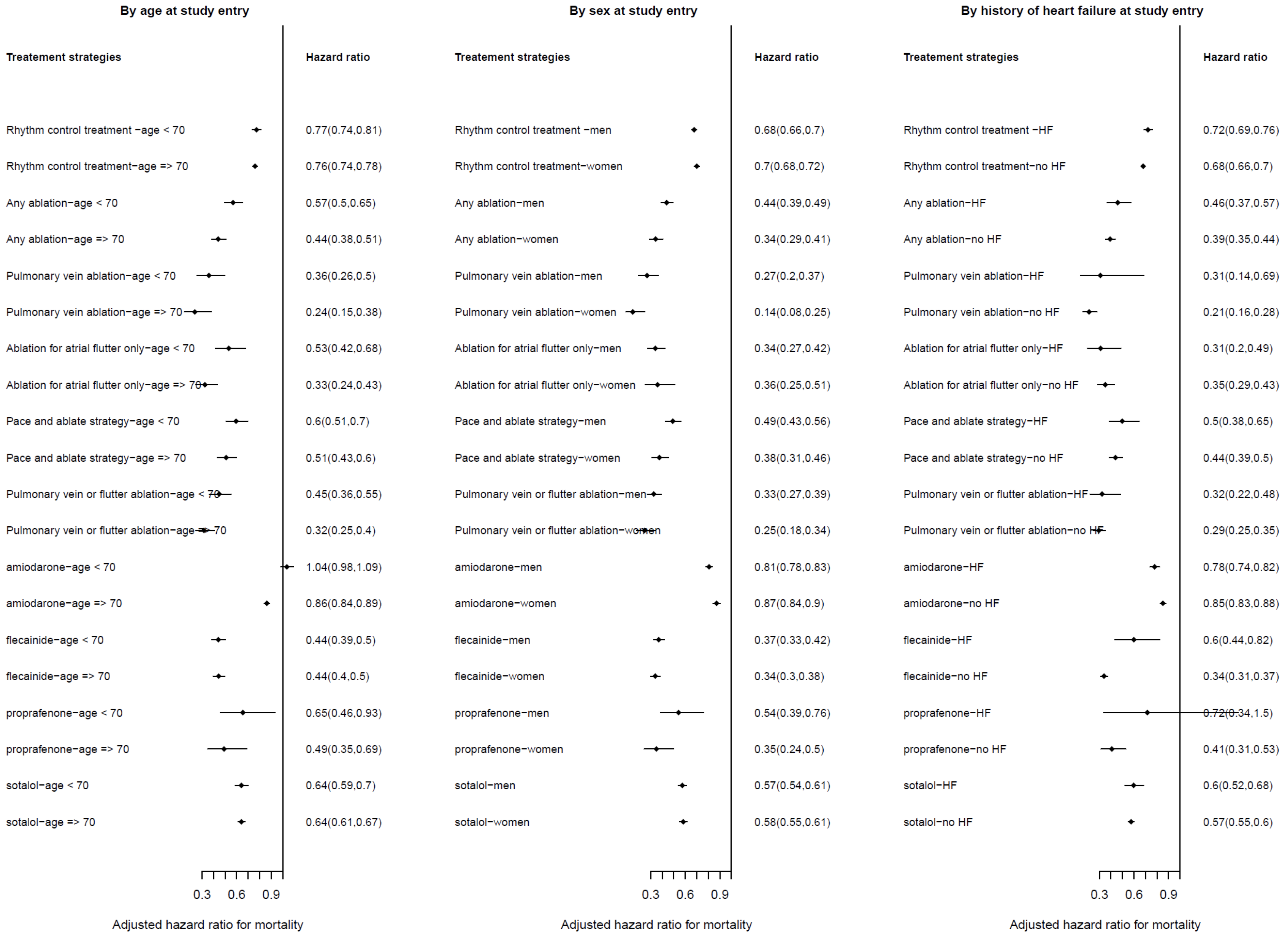

Figure S2: Multivariate-adjusted Cox regression analyses for mortality risk by rhythm control treatment strategies by patient subgroups.

Table S7. Mortality at 5 years per treatment option

Treatment	Mortality at 5th year of follow-up (%)
No rhythm control	47.3 (47,47.61)
Rhythm control	21.6 (21.1,22.16)
Amiodarone	26.7 (26,27.45)
Flecainide	5.2 (4.6,5.99)
Proprafenone	6.8 (4.6,10)
Sotalol	14.7 (14,15.53)
Any ablation	4.3 (3.6,5.03)
PVI	1.6 (1,2.51)
Flutter ablation	4.5 (3.5,5.89)
PVI or flutter ablation	3.3 (2.6,4.23)

Table S8. cause of deaths by treatment methods

Treatment	I: circulatory diseases	95% CI	C: neoplasms	95% CI	J: respiratory diseases	95% CI	K: digestive diseases	95% CI
Rhythm control treatment	4172 (45.8%)	(39.9, 40.5)	1749 (19.2%)	(16.4, 16.9)	1454 (16%)	(16.8, 17.3)	331 (3.6%)	(4.1, 4.4)
No rhythm control treatment	32032 (41.9%)	(43.3, 45)	13504 (17.7%)	(17.7, 19)	13216 (17.3%)	(15.3, 16.5)	3472 (4.5%)	(3.3, 4)
Any ablation	195 (47.1%)	(41.1, 48.5)	77 (18.6%)	(17.1, 23.1)	60 (14.5%)	(12, 17.3)	16 (3.9%)	(2.1, 4.8)
Pulmonary vein ablation	27 (55.1%)	(38.7, 59.3)	7 (14.3%)	(12.9, 29.7)	6 (12.2%)	(4.3, 16.7)	2 (4.1%)	(0.2, 7.2)
Ablation for atrial flutter only	48 (42.9%)	(33.9, 48)	23 (20.5%)	(18.2, 30.6)	21 (18.8%)	(11.9, 22.8)	5 (4.5%)	(1.8, 7.9)
Pace and ablate strategy	149 (48.7%)	(41.3, 49.9)	56 (18.3%)	(15.7, 22.5)	38 (12.4%)	(10.8, 16.8)	12 (3.9%)	(1.7, 4.9)
Pulmonary vein or flutter ablation	66 (45.2%)	(37.8, 50)	28 (19.2%)	(17.6, 28)	26 (17.8%)	(10.9, 19.8)	6 (4.1%)	(1.6, 6.3)
amiodarone	3335 (47.4%)	(44.8, 46.7)	1253 (17.8%)	(16.4, 17.9)	1192 (16.9%)	(16.1, 17.6)	244 (3.5%)	(3.1, 3.8)
flecainide	173 (33.7%)	(29.9, 36.2)	142 (27.6%)	(23, 28.9)	94 (18.3%)	(15, 20.1)	23 (4.5%)	(2.9, 5.7)
proprafenone	28 (43.8%)	(30.3, 49.9)	24 (37.5%)	(21.5, 39.9)	5 (7.8%)	(5.5, 18.3)	1 (1.6%)	(0.2, 6.8)
sotalol	1036 (42.7%)	(40.2, 43.3)	572 (23.6%)	(19.5, 22.1)	292 (12%)	(11.6, 13.7)	100 (4.1%)	(3.6, 4.8)
